# Medical Opinion and Movement

**Published:** 1911-11-04

**Authors:** 


### MEDICAL OPINION AND MOVEMENT.

### Arterio-sclerosis.

It is known that arterio-sclerosis shows a preference for certain arteries or even certain portions of these vessels. In the case of the aorta, changes are usually most marked on the ascending and transverse portions. According to Albrecht, the arterial segments most subject to chronic inflammatory changes are those which by insufficient means of fixation are most exposed to the effects of systolic distension, or, again, those which at the moment of distension suffer compression against a subjacent hard tissue, as in the case of the vertebral artery. According to Dr. Obendorfer, however, the factors at work are rather different and more complex. An account of his views appears in the *Deutsche Archiven für Klinische Medizin*. By examining the arteries at a series of autopsies on subjects of more or less advanced years he finds that the phenomena of arterio-sclerosis are most marked in those arteries or portions of arteries that are fixed, such as the internal carotid in the carotid canal, whereas those portions that are subjected to the movements of the muscles or joints of the body are least affected. In the case of the vertebral artery, the portion in the cranium is found rigid, with calcareous deposits, but the part passing through the vertebral canal and subjected to the movements of the head remains supple and free from sclerotic changes. Similarly in the case of the main arteries of the lower extremity, the parts most affected by sclerotic changes are the common iliac and upper part of the external iliac, the femoral from the origin of the deep femoral to the popliteal, and the tibial and peroneal arteries. On the other hand, the least affected portions are the lower part of the external iliac, upper portion of the femoral, and the popliteal arteries. These last portions are exposed to a sort of natural massage by the movements of the limbs, and so, according to the author, get increased nutrition and circulatory changes. The author suggests, therefore, that in such cases the condition of sclerosis might be inhibited or at least kept stationary by appropriate massage.

### Korff on Scopomorphine.

For ten years Korff has been experimenting with and advocating scopolamine as an adjunct to, or substitute for, inhalation anæsthesia. Since most of the British authors who have taken up this method have done so only within the last four years, the opinions of an authority who has published seven papers on it since 1900 must carry due weight. His latest contribution appears in the *Australasian Medical Gazette*, and contains several points of interest. In passing, it may be mentioned that Korff upholds the identity of scopolamine and hyoscine, which some regard as isomeric but not quite identical. He goes on to remark on the widely varying susceptibility of individuals to the drug, an indication for "divided dosage," and careful observation of the effect of one dose before the next is given. Korff admits that to aim at complete narcosis by scopomorphine is dangerous, and that in principle narcosis by injection is a retrogression, not an advance, on anæsthesia by inhalation. For goitre and similar operations for which general anæsthesia is dangerous he prefers scopomorphine alone, though he notes that there may be a "violent reaction" when the first incision is made. He divides 9/20 gr. of morphine and 1/55 gr. of scopolamine hydrobromide into three doses: one is given two hours and a half before operation, one an hour and a half before, and one half an hour before, but the condition of the patient governs the second and third injections, which are not always necessary. This dose is the maximum which he considers justifiable, and it is made up in sterile phials for him by the firm of Riedel; thus prepared it will keep indefinitely, as he has proved by chemical and by physiological tests. He lays great stress on the necessity for absolute purity of the drugs, the most important contamination being apoatropine.

### Oxalæmia.

Drs. Loeper and G. Béchamp have reported to the Société Médicale a number of observations on patients in whom they have shown an increase in the oxalic acid content of the blood. They have found that in such cases this oxalæmia is most commonly, though not invariably, proportional to the oxaluria. Patients in whom this oxalæmia is met with in its most marked form are diabetics, the gouty, those subject to intestinal and renal lithiasis, and also those suffering from rheumatism, neuralgia, and badly defined mucorrhœa. A portion of this excess of oxalic acid is eliminated by the kidneys, but other portions are got rid of by the bowel, the stomach, and even the respiratory organs. Some may become fixed in the tissues, especially in those of the nervous system, but a certain quantity is destroyed by being split up into carbonic acid and carbon monoxide. Clinically oxalæmia is accompanied by renal or intestinal lithiasis; by gastric, respiratory, and asthmatic crises; by nervous, articular, and vascular phenomena, and by blood changes. Treatment is partly preventive and partly consists in endeavouring to neutralise, destroy, and eliminate the acid already present in the organism.

### Pneumococcal Pulmonary Œdema.

Drs. Caussade and Milhit have published in the *Gazette des Hôpitaux* an interesting review of pulmonary œdema due to pneumococcal infection. They recognise, first of all, an acute type in which pulmonary œdema may be the only indication of a pneumoccocal invasion. At times acute œdema of the lung or larynx may be the first sign of a pneumococcal septicæmia, which later will declare itself by some other sign or localisation—*e.g.* pneumonia, arthritis, etc. Or, again, acute œdema may declare itself in the course of a pneumonia, either without any apparent reason, or as the result of some co-existing cardiopathy. By the side of the acute forms there exist subacute ones, in which the pneumococcus is the predominating organism found when the sputum is examined. In the same way, in the pulmonary œdema of the death-agony and in that of the aged, the authors have found virulent pneumococci present in the serous effusions. In the case of the acute forms of œdema the pneumococcus is often the sole provocative agent; more frequently the infection is a mixed one, infection being added to some toxic condition. In subacute cases, in addition to the infectious cause there may be extraneous factors present, such as chloride retention and suprarenal activity.

### Resumption of Breast-feeding after Weaning.

In *La Gynécologie*, Dr. R. Willette calls attention to the fact that it is possible to cause a resumption of the secretion of milk in a mother whose child has for some reason or other been weaned some months before. In one of the cases cited by him the mother had weaned the child after the first month. Three months later, as the child was in a deplorable condition, it was determined to make an endeavour to replace it at the breast, although no milk could be procured from the mother. In a day or two a few drops of milk were secreted, then a few grammes daily, and at the end of a fortnight the amount yielded in the twenty-four hours totalled over three ounces and a half. A fortnight later no less than ten ounces were secreted daily, after which time the yield was over twenty-eight ounces. The author insists on the necessity for patience and perseverance, but maintains that with these failure is impossible. The only certain lactagogue is the infant's suction, and the stronger the infant the quicker is the flow of milk re-established. It may in some cases be possible, and especially when commencing to re-establish the flow, to help matters by a judicious use of the breast-pump and massage of the breast. He finds that lactagogues of the type of placental extract have little influence other than possibly a moral one on the patient. The author's work is of interest, since in many cases natural feeding may have been interrupted merely because of some fleeting cause, such as an attack of appendicitis, salpingitis, etc.

### The Pathology of Senile Cataract.

Frenkel, in *Les Annales d'Oculistique*, gives an interesting study of the pathology of subcortical senile cataract, based to a large extent upon the result of his own researches. He finds that the condition is the result of both local and general causes. The first consist in functional alteration of the epithelium of the ciliary body, which allows of the passage of specific toxic substances. The general causes are due to the defective destruction or elimination of the same products. Renal insufficiency is the factor which has been studied most. The toxic substances belong to the class of autotoxins, products of the cellular activity of the body, and resulting especially from the destruction of the cells of the crystalline lens and the ciliary body which are functionally interdependent. They probably have a specific action on these bodies, but none upon the other body organs. There may be an hereditary predisposition to the accumulation of cytotoxins sufficient to produce cataract. Arterio-sclerosis, whether local or general, is not the cause of cataract. Treatment consists in the endeavour to destroy or neutralise these cytotoxins by the introduction into the system of specific anticytotoxins. These facts seem to be confirmed by clinical experience, and explain the appearance of cataract in the course of various general affections and congenital lesions.

### Ipecacuanha in Tropical Hepatitis.

The value of powdered ipecacuanha in comparatively large doses in the treatment of tropical dysentery, especially in its early stages, has been established beyond dispute, but it is less recognised that the drug is sometimes equally beneficial when the symptoms are hepatic after the intestinal symptom has subsided. Short of the production of actual abscesses in the liver it is not at all uncommon to find after a mild attack of dysentery that there is a tender swelling of the organ which can only be described as acute hepatitis or acute congestion of the liver, and which in not a few instances subsides in a longer or shorter time without going on to the production of an actual abscess. There is evidence to show that even when there are no longer any intestinal symptoms the administration of powdered ipecacuanha goes far towards accelerating the subsidence of the hepatic symptoms and therefore, one may conclude, towards minimising the risk of the development of an abscess of the liver after dysentery. Some authorities combine 10 grains of tannic acid with the ipecacuanha, upwards of 30 grains of the latter being given night and morning.

### Chronic Ovaritis.

The diagnosis of chronic ovaritis, once so common, has fallen into discredit with gynæcologists of late years; and there is no doubt that it did serve as a cloak for much ignorance and was greatly misused. Dr. J. S. Young, in a Manchester thesis, published in the *Medical Chronicle*, considers this lesion in detail. He concludes that chronic idiopathic, or primary, ovaritis oes not exist apart from tuberculosis or syphilis. All inflammations of the ovary are secondary to uterine or tubal lesions, or acute infections, toxic diseases, etc. Definite increase in the connective tissue over and above that which occurs within the limits of the normal is probably due to true hypertrophy or hyperplasia, rather than to chronic ovaritis. Periovaritis may exist, even to embedding the whole organ in dense adhesions, without the ovary exhibiting any changes other then physiological; so that a well-marked periovaritis is no criterion *per se* of chronic ovaritis. No symptom or group of symptoms is of any real service in arriving at the diagnosis of chronic ovaritis, for none is characteristic of the condition.

